# Effect of nystagmus on VEP-based objective visual acuity estimates

**DOI:** 10.1038/s41598-024-66819-y

**Published:** 2024-07-22

**Authors:** Elisabeth V. Quanz, Juliane Kuske, Francie H. Stolle, Michael Bach, Sven P. Heinrich, Michael B. Hoffmann, Khaldoon O. Al-Nosairy

**Affiliations:** 1 Ophthalmic Department, Otto-Von-Guericke University Magdeburg, Magdeburg, Germany; 2 Eye Center, Medical Center – University of Freiburg, Freiburg, Germany; 3 Faculty of Medicine, University of Freiburg, Freiburg, Germany; 4 Center for Behavioral Brain Sciences Magdeburg, Magdeburg, Germany

**Keywords:** VEP, Nystagmus, Visual acuity, Microperimetry, Object vision, Visual system, Retina

## Abstract

In order to determine the effect of nystagmus on objective visual acuity (VA) estimates, we compared subjective (VA_psych_) and objective (VEP, VA_VEP_) VA estimates in participants with nystagmus. For this purpose, 20 participants with nystagmus (NY) caused by idiopathic infantile nystagmus, albinism, achiasma or acquired nystagmus were recruited in this study. Estimates of BCVA (best corrected visual acuity) were determined psychophysically (VA_psych_; FrACT, Freiburg visual acuity test) and electrophysiologically (VA_VEP_; EP2000) according to ISCEV (International Society of Clinical Electrophysiology of Vision) guidelines. For each participant the eye with the stronger fixation instability [Nidek microperimeter (MP-1), Nidek Instruments] was included for further analysis. VA_psych_ vs VA_VEP_ were compared via paired t-tests and the correlation of the difference between VA_psych_ and VA_VEP_ (∆VA) vs the degree of fixation instability was tested with Pearson correlation (r). We found VA_VEP_ to be better than VA_psych_ [by 0.12 Logarithm of the Minimum Angle of Resolution (logMAR); mean ± standard error (SE) of VA_VEP_ vs VA_psych_: 0.176 ± 0.06 vs. 0.299 ± 0.06, P = 0.017] and ∆VA to be correlated linearly with the degree of fixation instability (r^2^ = 0.21,p = 0.048). In conclusion, on average we report a small VA overestimation, around 1 line, for VA_VEP_ compared to VA_psych_ in NY. This overestimation depended on the magnitude of the fixation instability. As a rule of thumb, a reduction of the fixation probability in the central 4° from 100 to 50% leads on average to a VA_VEP_ overestimation of around 0.25 logMAR, i.e. 2.5 lines.

## Introduction

Subjective visual acuity testing is a ubiquitous routine tool for everyday clinical practice in ophthalmology and often the first step to check the integrity of retinal function and subsequent structures of the visual system. Critically, this measure of visual acuity (VA_psych_) is challenged by the subjective nature of the responses, depends on the compliance of the participants, and is hence vulnerable e.g. to malingering^1^. This creates a need for an objective measure of visual acuity, which might be met by visual evoked potential (VEP) based visual acuity estimates (VA_VEP_). In fact, previous research in this field has demonstrated the value of this approach^2^. However, limits to the applicability of VA_VEP_ naturally are expected for certain conditions, which might be particularly prone to systematic VA misestimations. The identification of such conditions is instrumental to avoid the misinterpretation of a patient's VA_VEP_ results. Within this context, it is uncertain whether VEP-acuity estimations maintain their validity in the presence of nystagmus.

As a matter of course, VEP-recordings depend on the capacity of a visual stimulus to drive responses in the visual cortex. One critical parameter is the spatial frequency of the stimulus, as only stimuli that can be resolved will generate responses. It is this dependence of the VEP on spatial frequency of the visual stimulus that is exploited for the estimation of the resolution limit in acuity-VEP paradigms. Specifically, these paradigms target the detection of the spatial frequency limit beyond which stimuli fail to elicit a response^2^. Another critical parameter for VEP recordings is the stimulation mode. This includes the distinction of pattern-reversal vs pattern-pulse stimulation, which is of particular importance when targeting populations with nystagmus. Notably, nystagmus reduces pattern-reversal VEPs, while VEPs to pattern-pulse stimulation are relatively spared^3,4^. Acuity-VEP paradigms are often based on pattern-pulse stimulation^2^. As a consequence, responses from participants with nystagmus are expected to be only little affected, preserving the validity of VEP-acuity estimates in nystagmus, or might even be overestimated. In fact, some reports do suggest the potential of an overestimation of VEP-acuity for nystagmus^5–7^. A systematic assessment of this issue with a contemporary approach to determine VEP-acuity is at present missing. At the same time, however, as nystagmus is a common feature in patients with low vision, it is of critical importance for clinical applications, whether the validity of VA_VEP_ measures is confounded by nystagmus.

We aimed to investigate the validity of VA_VEP_ in the presence of nystagmus by employing a VEP paradigm that is based on patten-pulse stimulation^8^. Specifically, we compared VA_psych_ and VA_VEP_ estimations in a cohort of patients (NY) with nystagmus due to different etiologies. To accurately determine the effect of eye motion on VA_VEP_ misestimation, fundus imaging was employed to quantify fixation instabilities. We hypothesized that VA_VEP_ might be overestimated compared to VA_psych_ in NY particularly, if fixation instabilities are pronounced.

## Methods

This prospective observational study was conducted at the ophthalmic department of the Otto-von-Guericke University (OVGU), Magdeburg, Germany.

### Participants

Participants were included after an ophthalmological examination: 20 participants with nystagmus (NY; 9 females; age (mean, range across all participants): $38, 20-64\text{y}$) due to the idiopathic infantile nystagmus syndrome (n = 10), albinism (n = 6), achiasma (n = 1), or acquired nystagmus (n = 3), as detailed in Table 1. Optic nerve misrouting in albinism^9,10^ and achiasma^11,12^ was confirmed via the misrouting VEP^13,14^, a VEP method detailed further in previous publications^9,15,16^. To study the effect of nystagmus on measures of VA, only the individual's eye with stronger fixation instability determined by microperimetry test (see below) was included in the analysis. Exclusion criteria were epilepsy, dizziness, neurological diseases unrelated to nystagmus and any eye diseases affecting visual function, e.g., diabetic retinopathy.

**Table 1:** Overview of participants' including characteristics of each participant's selected eye, on the basis of stronger fixation instability.

ID	Group	Sex	Age [years]	Nystagmus type	Stereopsis*	VEP	BCVA [logMAR]	Eye†	Fixation ± 2° [%]
BJA815	INS	m	21	J/H	Yes	–	0.12	OS	91
MFY773	INS	f	24	J/H	No	–	0.24	OS	83
ENH995	INS	f	21	J/H	No	–	0.54	OD	42
XAY182	INS	f	42	J/H	No	-	0.41	OD	86
SXB794	INS	f	60	J/H	No	-	-0.03	OD	99
JDG458	INS	m	29	P/H	No	-	0.32	OS	37
WQE170	INS	m	23	P/H	Yes	-	0.06	OD	n/a
SUQ660	INS	m	34	J/H	Yes	-	-0.15	OD	99
PEP763	INS	f	56	J/H	No	-	0.32	OS	99
DKX711	INS	m	51	J/H	Yes	-	0.28	OS	74
TGY248	AN	f	30	J/V	Yes	-	-0.08	OD	96
TIO945	AN	m	38	J/H	Yes	-	0.05	OD	98
UFX538	AN	f	42	J/H	Yes	-	-0.07	OD	99
PYV946	AL	f	23	J/H	No	+	0.47	OD	74
JTE807	AL	m	51	J/H	No	+	0.73	OS	91
GRV905	AL	m	63	J/H	No	+	0.69	OS	99
PNJ290	AL	m	64	J/H	No	+	0.69	OS	86
SLP201	AL	f	20	J/H	No	+	0.61	OS	36
SDN948	ACH	m	22	J/H	No	+	0.27	OS	88
XZE409	AL	m	40	J/H	No	+	0.57	OD	43

BCVA [logMAR]: best corrected visual acuity; VEP: misrouting visual evoked potential; “ + ”/ “–” indicates presence/absence of optic nerve misrouting (negative/positive correlation coefficient between both eyes' inter-hemispherical activation difference); INS: idiopathic infantile syndrome (excluding albinism); AN: acquired nystagmus (causes: Arnold-Chiari Syndrome, pons bleeding, hydrocephalus shunt surgery); AL: albinism; ACH: achiasma; Nystagmus type: J: jerk, P: pendular, H: horizontal, V: vertical; f: female; m: male; n/a: not available. *Stereopsis test using Lang test. †Eye with stronger fixation instability.

### Microperimetry—Fixation stability

Fixation stability of participants within 2° and 4° was quantified by tracking fundus-motion at 25 Hz with a fundus-controlled microperimeter (MP-1 microperimeter, Nidek, Padua, Italy), for an epoch of 30 s, where participants were asked to fixate a central target. Eyes with stronger fixation instability, using fixation proportion within the central ± 2°, were selected for the analysis. See Table 1 for characteristics, including fixation instability and BCVA, of each participant's selected eye.

### Subjective VA estimation

Estimates of best corrected visual acuity (BCVA) for each eye were determined psychophysically (VA_psych_) using the “Freiburg Visual Acuity and Contrast Test” [FrACT; VA_psych_,^17^] applying Landolt Cs at a viewing distance of 114 cm (as for the VA_VEP_ estimation). Every measurement was performed twice to reassure the validity of the measurements.

### Objective VA estimation

Objective VEP-acuity testing (VA_VEP_) was estimated according to International Society for Clinical Electrophysiology of Vision (ISCEV) standards^18^ and followed the procedure described previously^8^. Briefly, steady state (ss)-VEPs were recorded using pattern-pulse stimulation at 7.5 Hz as detailed in Table 2. The ssVEPs were Fourier analyzed. For each spatial frequency (SF [cpd] = 1/√2 × check size) the response magnitude at the stimulation frequency (7.5 Hz) and a noise estimate, the average of the response of the two neighboring frequencies (6.5 and 8.5 Hz) were obtained to determine the ‘true’, i.e. noise-corrected, amplitude A^*^(SF)^19–21^ and the significance-level of the response *p*(SF)^21^. A stepwise heuristic algorithm^8^ was applied to calculate the upper SP where the log amplitude response extrapolated to zero, i.e., SF_0_. SF_0_ was converted to VEP acuity [decimal VA_VEP_ = SF_0_/17.6 cpd, which corresponds to logMAR VA_VEP_ = log(SF_0_/17.6 cpd)]. For all participants tested, the heuristic algorithm produced an estimated VA_VEP_ (100% testability).

**Table 2:** Overview of electrophysiological recording parameters.

	VEP-based VA
Recording Device	EP 2000 Evoked potentials system ^22^
Monitor	Monochrome CRT monitor (MDG403, Philips; P45 phosphor; 75 Hz)
Stimulus	Checkerboard
Ambient light	Dimly lit room
Mean luminance	50 cd/m^2^; 40% contrast
Size of stimulus	For BCVA < 0.3 [logMAR]: Six logarithmically equidistant steps from 0.048° to 0.385° For BCVA values > 0.3 [logMAR]: A different sequence of check sizes was utilized: **0.09—0.8°**
Stimulation	steady-state brief onset pattern stimulation (7.5-Hz, 40 ms on, 93 ms off)
Electrode placement	Laplace montage ^23^: Gold-cup electrodes at Oz, LO and RO, referenced to FPz (according to the international 10–20 System)
Recording setting	Signals amplified 100 K times the VEP signals & band pass filtered them (0.3, 70 Hz)
Artefact rejection	± 90 µV threshold
Eye	Monocular recording
Viewing distance	114 cm
Repetitions	A-B-B-A*
Fixation control	Random digits from 0–9 in the center of the screen & verbal feedback by participants
Processing	Responses digitally filter with low-pass cutoff of 40 Hz
Reporting	Decimal VEP-based BCVA ^8^

Amplifier: Grass Model 15, Astro-Med Inc., West Warwick, RI, USA.

For further calculation of Correlation coefficient, [see^15^ and text].

See further info of VA-VEP estimations^8^. *Repetitions trials were averaged.

### Analysis and statistics

Only one eye of each participant was included, namely the eye with the stronger fixation instability. As the data passed the Shapiro Wilk test for normality, parametric statistical testing was applied. The VA_psych_ vs VA_VEP_ estimates were compared using a paired t-test. Pearson correlation (r) was applied to test whether the difference between VA_psych_ and VA_VEP_ (∆VA) correlated with the degree of fixation loss within 4° determined by Pearson correlation (r).

### Ethical approval

The study was conducted according to the guidelines of the Declaration of Helsinki, and approved by Ethics Committee of Faculty of medicine, Otto-von-Guericke University, Magdeburg (153/18).

### Informed consent

Informed consent was obtained from all individual participants included in the study.

## Results

**Figure 1:**
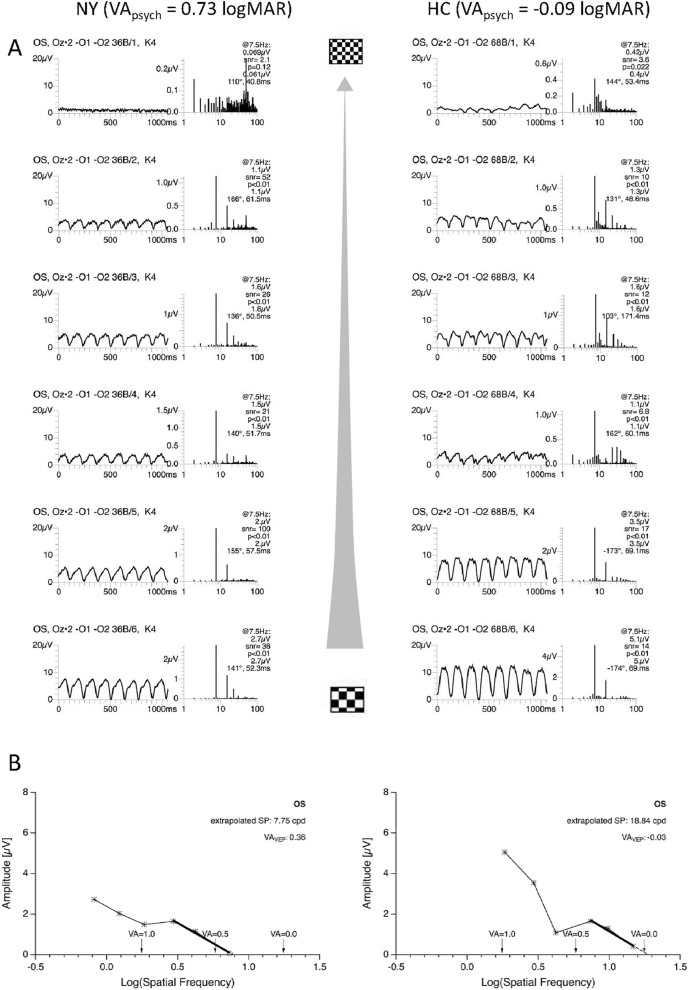
VEP data from two examples. Visual evoked potential visual acuity (VA_VEP_) and subjective VA (VEP_psych_) are given for two individuals, i.e., a healthy control (HC) and a participant with nystagmus (NY). A) VEP-results underlying the estimation of VA_VEP_. For each participant, NY (JTE807) and a HC (OEX914), two panels are given. The left panel depicts the raw VEP traces for the different check sizes ranging from 0.046° to 0.370° . The right panel depicts the power spectrum and the respective significance of the response’s amplitude at 7.5 Hz, i.e., the stimulation frequency, which enter the spatial frequency tuning curve. B) Spatial frequency tuning curve and VA_VEP_ estimate for NY and HC. VA_VEP_ is derived from the extrapolated spatial frequency (SF) limit determined from the regression line (strong black line) according to Bach et al.^8^, as detailed in Methods. Significant responses are denoted with an asterix, non-significant with open symbols. For NY VA_psych_ and VA_VEP_ (0.73 vs 0.36) match less closely than for HC (− 0.09 vs − 0.03).

**Figure 2:**
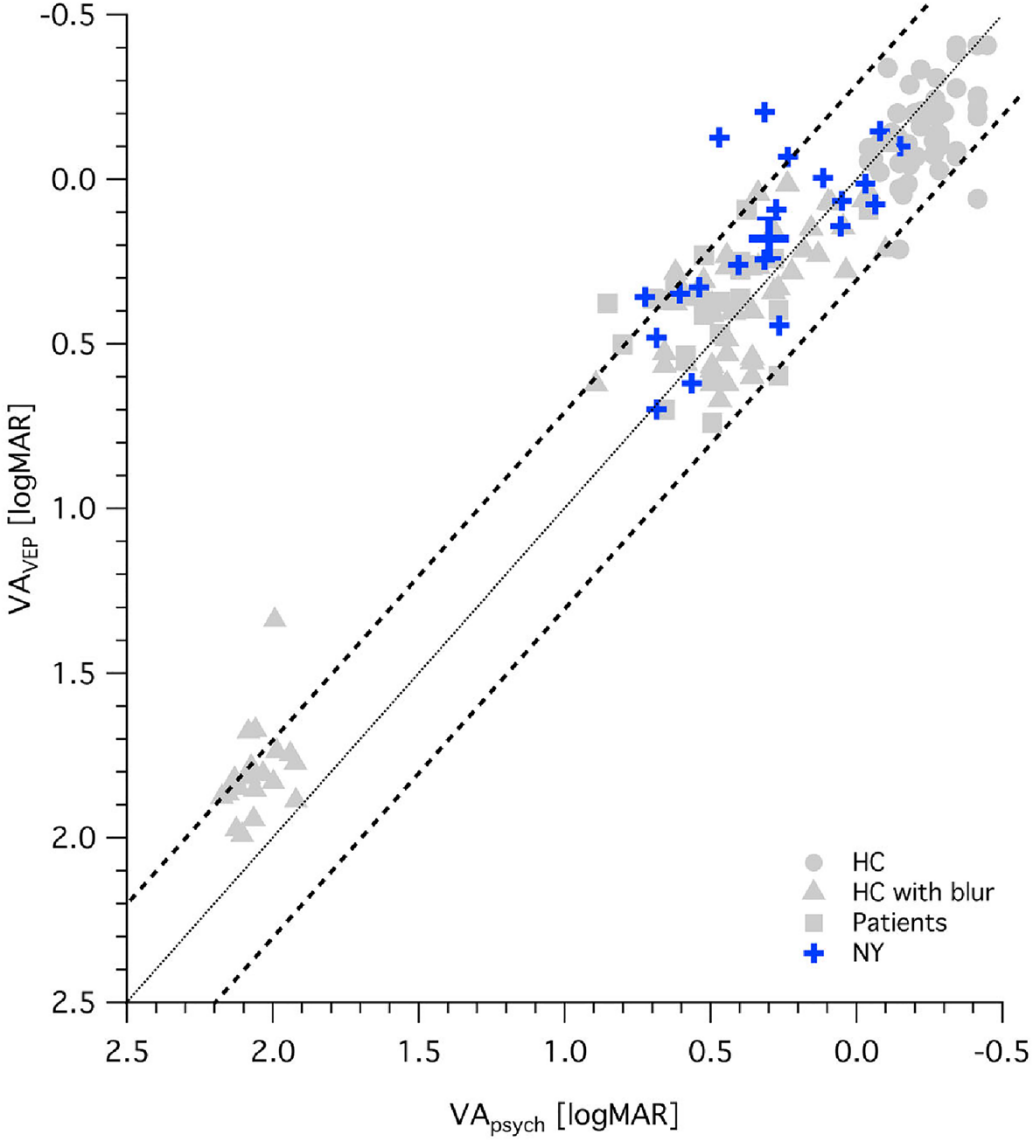
Objective vs subjective visual acuity. Participants with nystagmus [large symbol: mean ± SEM for VA_VEP_] are given in comparison to previously published data from participants without nystagmus^8,24^). For our nystagmus cohort, 4 out of 20 eyes fell above the 95% CI established in Bach et al. 2008, indicating an acuity overestimation.

For a qualitative overview of the VEP-recordings obtained, VEP traces for a representative participant with nystagmus (NY) and a healthy control (HC) are juxtaposed in Fig. 1A together with the acuity estimation (Fig. 1B) according to the heuristic algorithm published previously^8^. The group's data (20 participants with nystagmus, for each individual's eye with stronger fixation instability) are depicted in Fig. 2 (cyan symbols). For comparison, results previously reported for participants without nystagmus by Bach et al. 2008 and Hoffmann et al. 2017 are added (gray symbols). Overall, there was a significant overestimation of VA_VEP_ vs VA_psych_ for nystagmus by on average − 0.12 logMAR (mean ± SE of VA_VEP_ vs VA_psych_: $0.18\pm 0.06\text{ vs} \, 0.30\pm 0.06,\text{ p}= 0.017$). Still, 16 (80%) of the 20 eyes were within the 95% confidence interval determined in Bach et al. 2008 for participants without nystagmus.

**Figure 3:**
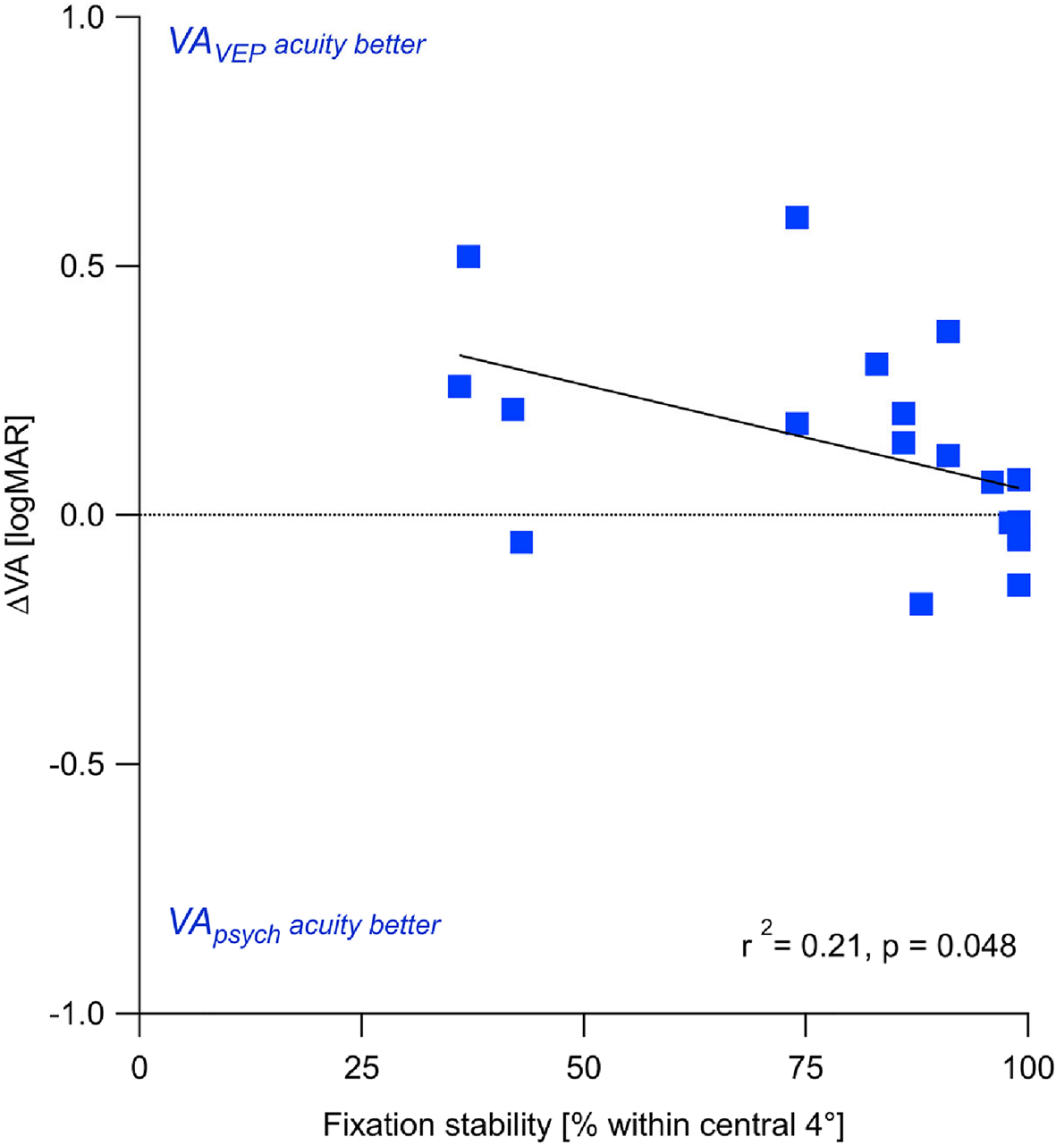
Correlation of VA_VEP_–VA_psych_ differences (∆VA) and fixation stability in NY. The significant correlation indicates that VA_VEP_ is overestimated for low fixation stability, i.e. more pronounced nystagmus. Fixation stability explains 21% of the variance.

To test whether the VA_VEP_ overestimation in nystagmus was associated with nystagmus severity, we employed a measure of fixation instability (fixation proportion within the central ± 2°) as a surrogate measure of nystagmus severity. We tested the correlation of the acuity differences (∆VA = VA_VEP_–VA_psych_) with fixation instability. In fact, there was a weak, albeit significant correlation between fixation instability and the $\Delta$ VA (r^2^ = 0.21, p = 0.048; Fig. 3). Consequently, 21% of the variance in the data can be attributed to the strength of the participants' fixation instability.

## Discussion

We tested whether nystagmus affects the relationship of VA_VEP_ and VA_psych_. In our cohort of 20 eyes, we report an overestimation of visual acuity, i.e., better VA, for nystagmus by around 1 line, i.e., 0.12 logMAR. The difference between VA_VEP_ and VA_psych_ correlated significantly with the degree of fixation loss, indicating that the VA_VEP_ estimates were particularly affected by higher degree of nystagmus.

VA_VEP_ offer a complementary or alternative option to assess VA, in cases where VA_psych_ appears questionable. However, as recently reported^2^, consistency between VA_VEP_ and VA_psych_ is dependent on the etiology of visual dysfunction and acuity loss. Comparable VA_VEP_ and VA_psych_ reductions were reported in media opacities or retinal pathologies, while a VA_VEP_ and VA_psych_ mismatch is more likely in optic nerve, neurological diseases or amblyopia. For patients, where the stimulation is subject to retinal image slip, due to nystagmus, the matter was still unresolved. For our cohort we demonstrate the effect to be minor on average, i.e. an overestimation of around 1 line, but that stronger effects are more likely for higher degree of nystagmus-related fixation loss. Previous research on NY as a separate disease entity was up to now limited as reflected by a few studies from the 80-ies and 90-ies: In a small number of NY patients (n = 5) VA_VEP_ was reported to be poorer than VA_psych_ by 0.15 logMAR^5^. In a cohort of 14 NY children, Gottlob et al.^6^ found slightly and non-significantly better estimates of VA_VEP_ (Sinusoidal sweep VEP, 0.48 log min arc) than VA_psych_ (recognition VA using Allen picture cards, 0.51 log min arc) and VA_VEP_ vs VA_psych_ not to be correlated. Westall et al.^7^ also reported a non-significant trend of a VA_VEP_ overestimation by 0.09 logMAR compared to acuity-card VA in a group of NY children. Different stimulation paradigms, sample size, and study design of these earlier studies, might account for the discrepant findings between studies.

### Practical considerations, limitations and outlook

In this study, we assessed the VEP-based VA estimation in comparison to subjective VA measures in an important disease cohort, nystagmus. We highlighted the importance of taking fixation stability in consideration when evaluating VA_VEP_ in nystagmus, as 21% of the variance in the data can be attributed to fixation instability. As a rule of thumb, a reduction of the fixation probability in the central 4° from 100 to 50% leads on average to a VA_VEP_ overestimation of around 0.25 logMAR, i.e. 2.5 lines.

At present, the mechanisms that might mediate this overestimation of VA_VEP_ compared to VA_psych_ are unclear. They might be associated with different stimulation schemes used for the VA estimation, as pulsed patterns are used for VA_VEP_ as opposed to continuous stimulation for VA_psych_. As an alternative to a methodological cause, there might be a physiological cause. E.g., an association of nystagmus with other pathophysiologies that might lead to VA overestimation, e.g. amblyopia^25^. Further research is needed to address this issue. Additional insights into the underlying mechanisms might be uncovered in studies that employ fixation-monitoring via eye-tracking during the VEP recordings for a quantitative account of the fixation instabilities and ultimately correct for eye-movements that during VEP recording. Moreover, investigations that specifically address the dependence of VA_VEP_ -overestimation in nystagmus on etiology, ocular co-morbidity and type and strength of nystagmus might be of promise to specify the clinical implications of the influence of nystagmus on VEP-acuity.

## Conclusion

This study reported a slight but significant overestimation of VA_VEP_ compared to VA_psych_ in the presence of nystagmus. The differences between objective and subjective VA estimates depended on the magnitude of fixation loss, i.e., higher magnitude of instability leads to higher differences between objective and subjective VA measures. This dependence needs to be taken into account when evaluating VA_VEP_ estimates in nystagmus, specifically when fixation instabilities are pronounced.

## Data Availability

Data are available upon request. Please contact the corresponding author, MBH, for data requests.
